# Is adolescent multiple risk behaviour associated with reduced socioeconomic status in young adulthood and do those with low socioeconomic backgrounds experience greater negative impact? Findings from two UK birth cohort studies

**DOI:** 10.1186/s12889-021-11638-3

**Published:** 2021-09-03

**Authors:** Laura Tinner, Caroline Wright, Jon Heron, Deborah Caldwell, Rona Campbell, Matthew Hickman

**Affiliations:** Population Health Sciences, Bristol Medical School, University of Bristol, BG3 Oakfield House, Bristol, BS8 2BN UK

**Keywords:** Adolescence, Socioeconomic factors, Cohort studies, Inequalities, Multiple risk behaviour

## Abstract

**Background:**

Adolescent multiple risk behaviour (MRB) is associated with negative outcomes such as police arrests, unemployment and premature mortality and morbidity. What is unknown is whether MRB is associated with socioeconomic status (SES) in adulthood. We test whether adolescent MRB is associated with socioeconomic status (SES) in young adulthood and whether it is moderated by early life SES variables.

**Methods:**

Prospective cohort studies; British Cohort Study 1970 (BCS70) and Avon Longitudinal Study of Parents and Children (ALSPAC), born in 1991–1992, were used and two comparable MRB variables were derived. Logistic regression was used to determine the association between MRB and young adult SES. The moderating effect of three early life SES variables was assessed using logistic regression models with and without interaction parameters. Evidence to support the presence of moderation was determined by likelihood ratio tests ≤*p* = 0.05. Multiple imputation was used to account for missing data.

**Results:**

Adolescents had a median of two risk behaviours in BCS70 and three in ALSPAC. Adolescent MRB was negatively associated with young adult SES (university degree attainment) in BCS70 (OR 0.81, 95% CI: 0.76, 0.86) and ALSPAC (OR 0.85, 95% CI: 0.82, 0.88). There was a dose response relationship, with each additional risk behaviour resulting in reduced odds of university degree attainment. MRB was associated occupational status at age 34 in BCS70 (OR 0.86 95% CI: 0.82, 0.90). In BCS70, there was evidence that maternal education (*p* = 0.03), parental occupational status (*p* = 0.009) and household income (*p* = 0.03) moderated the effect of adolescent MRB on young adult SES in that the negative effect of MRB is stronger for those with low socioeconomic backgrounds. No evidence of moderation was found in the ALSPAC cohort.

**Conclusions:**

Adolescence appears to be a critical time in the life course to address risk behaviours, due to the likelihood that behaviours established here may have effects in adulthood. Intervening on adolescent MRB could improve later SES outcomes and thus affect health outcomes later in life. Evidence for a moderation effect in the BCS70 but not ALSPAC suggests that more detailed measures should be investigated to capture the nuance of contemporary young adult SES.

**Supplementary Information:**

The online version contains supplementary material available at 10.1186/s12889-021-11638-3.

## Background

Risk behaviours such as excessive alcohol consumption, tobacco use, risky sexual behaviour and low physical activity commonly start in adolescence. Such behaviours can be detrimental to health and are costly to society [1]. There is evidence of the co-occurrence of risk behaviours, with individuals engaging in one behaviour being likely to adopt others [2]. Multiple risk behaviour (MRB) refers to the occurrence of two or more risk behaviours. MRB in adolescence has been shown to be associated with adverse health and social outcomes, such as unemployment [3], low educational attainment at GCSE level [4], getting in trouble with the police [5] and premature mortality and morbidity [1, 6]. Therefore, adolescence is increasingly acknowledged as a critical time in the life course to address health risk behaviours due to the likelihood that behaviours established here may have deleterious effects in adulthood.

Adolescence and young adulthood are also crucial in addressing health and social inequalities, as it is at this time that individuals can improve their life chances through education [7] and entry into the labour market [8]. Few studies predict from adolescence, with many studies concerned with exposures in early years, however, health risk behaviours initiated here are likely to be ‘major contributors to links between deprivation and inequality in later life’ [9]. Socioeconomic status (SES) is a construct used to assess inequalities, with commonly used measures including education, income or occupation [10]. Previous research has found early life or ‘origin’ SES to strongly predict SES in adulthood [11], however, examining SES as an outcome has seldom been done in longitudinal health research. Addressing this gap will increase our knowledge around how health risk behaviours contribute to inequalities later in life, through disturbing social circumstances in young adulthood [3, 12].

Given the lifestyle perspective of health inequalities, whereby it is hypothesised that less affluent individuals suffer a heavier burden of health issues due to the adverse behaviours they engage in [13], it is plausible to expect risk behaviours and socioeconomic status to interact across the life course. Risk behaviours may be more dangerous for some than for others due to differential social resources that may ‘buffer some of the costs of adolescent risk behaviour’ [14]. Therefore, accounting for potentially moderating factors in longitudinal data analyses on consequences is essential, although so far has been lacking [15]. Shackleton et al. [16] found there to be socially patterned changes in adolescent health behaviours such as tobacco smoking and obesity when examining two birth cohorts 30 years apart. This is consistent with the evidence that inequalities in the general population have increased for many health outcomes [17].

In this study, we used two prospective cohort studies, born 20 years apart to: (1) test the association between adolescent MRB and young adult SES as measured by university degree attainment in mid-twenties; and (2) assess whether early life SES moderates this association [18]. In doing so we aimed to illuminate the nature of the relationship between adolescent MRB on later life chances and determine whether this relationship was different for young people from differing socioeconomic backgrounds. Using two birth cohorts allowed us to test the use of the MRB variable in two populations and explore the associations in two different historical contexts.

## Methods

### Participants

#### 1970 British Cohort Study

The BCS70 sampled all births in Great Britain within 1 week in 1970 (*n* = 17,196) with 16,568 participants followed beyond birth. There have been nine sweeps of data collection up to age 46 years [19]. Although the initial cohort was representative of the UK at the time, those who dropped out of data collection sweeps are more likely to be male, from low socioeconomic backgrounds and have a single parent at birth [20]. The sample does not reflect the ethnic diversity of today’s population given the difficulty in recruiting immigrants to the sample at subsequent waves [19].

#### The Avon Longitudinal Study of Parents and Children (ALSPAC)

All children in the ALSPAC cohort were born to mothers residing in Avon in southwest England between April 1991 and December 1992 [21]. There were 14,541 pregnant mothers recruited, 14,062 live births and 13,988 (52% males and 48% females) singletons/twins still alive at 12 months old [22]. Although the original ALSPAC cohort was representative of the UK 1991 census, participants are more likely to be of white ethnicity (OR 3.85, 95% CI: 3.50, 4.24) and less likely to be eligible for free school meals (OR 0.46, 95% CI: 0.43, 0.50) than the national average [22].

### Measures

We identified SES and MRB measures from the two cohorts which were broadly comparable. All measures were self-reported. Some measures were more readily comparable than others and further details on the harmonisation process and derivation of the variables are available in Supplementary File 1.

#### Exposure: adolescent MRB

Table 1 contains the derivation of the health risk behaviors in each birth cohort. For BCS70, information on risk behaviour engagement was captured through a single questionnaire distributed by schools at age 16 years. For ALSPAC, information related to engagement in risk behaviour during adolescence was derived from a computer-based session during a clinic attended at age 15 and a later postal questionnaire at age 16.

**Table 1:** Derivation of MRB variables in both cohorts

Health risk behaviour	How variable was derived in BCS70	How variable was derived in ALSPAC
**Physical inactivity**	Young person (YP) has typically over the past year done sport in or out of school less than once a week (from an extensive list of sports). If young person said they took exercise last Saturday, they were included in the non-risk behaviour group.	Young person (YP) has typically over the past year exercised < 5 times per week.
**TV viewing**	–	YP spent 3 or more hours watching television on average per day across the week.
**Car passenger risk**	Young person has drunk and driven once or more AND/OR young person wears a seatbelt never, a few times or most times.	YP had been in a car passenger at least once in their lifetime where the driver (a) had consumed alcohol or (b) did not have a valid licence or (c) the YP chose not to wear a seat belt last time travelled in a car, van or taxi.
**Cycle helmet use**		If the YP reported that they had last ridden a bicycle within the previous four weeks and they had not worn a helmet on the most recent occasion.
**Scooter risk**		YP has driven a motorbike/ scooter off road or without a licence on a public road at least once.
**Criminal/Antisocial behaviour**	Young person reported that since the age of 10 at least one of the following offenses: Been questioned by police; let off with a warning; been arrested; formally cautioned; found guilty in court OR in the past year reported at least one of the following offenses: broken a window/smashed others property; stolen item from a shop; used physical force to get money; broken into a house to steal something; stolen a bike; broke into a cash dispenser; swore at a teacher; driven a car on the road underage; sold something shop lifted.	YP reported that at least once in the past year they had undertaken at least one of the following 7 offences:-carried a weapon; physically hurt someone on purpose; stolen something; sold illicit substances to another person; damaged property belonging to someone else either by using graffiti, setting fire to it or destroying or damaging it in another fashion; subjected someone to verbal or physical racial abuse; or been rude/rowdy in a public place.
**Hazardous alcohol consumption**	Young person has had four drinks or more in a row in the past two weeks AND has been really drunk in the last year.	In the past year had scored 8 or more out of 40 on the Alcohol Use Disorders Identification Test (AUDIT) indicating hazardous alcohol consumption. 10 questions based on drinking behaviour, scoring points for never (0 points), less than monthly (1 point), monthly (2 points), weekly (3 points) and daily/almost daily (4 points).
**Regular tobacco smoking**	Young person indicates smoking at least one cigarette per week.	Has ever smoked and is regularly smoking by currently smoking at least one cigarette per week.
**Cannabis use**	Young person reports having smoked cannabis 10+ times in the past year.	Those who reported using cannabis “sometimes but less often than once a week” or more regular use were classified as occasional users.
**Illicit drug/solvent use**	Young person has used any of the following drugs two or more times in the past year: cocaine; solvents; LSD; downers; uppers; heroin.	In the year since their 15th birthday, YP had either been a regular user (i.e. used five or more times) of one or more illicit drugs (excluding cannabis) including amphetamines, ecstasy, LSD, cocaine, ketamine or inhalants including aerosols, gas, solvents and poppers.
**Self-harm**	–	Young people who said they had purposely hurt themselves in some way in their lifetime.
**Penetrative sex before age 16**	–	YP reported having had penetrative sex in the preceding year and that they were under 16 at the time.
**Unprotected sex**	Young person reports method of contraception is “none”, “boy withdraws”, “use safe period” or “trust to luck”.	Penetrative sex without the use of contraception on the last occasion they had had sex in the past year

Eight comparable MRBs were used in both cohorts: physical inactivity; car risk; criminal/anti-social behaviour; hazardous alcohol consumption; regular tobacco smoking; cannabis use; illicit drug/solvent use; unprotected sex. The ALSPAC cohort had five additional MRBs: TV viewing; penetrative sex before age 16; scooter risk; cycle helmet risk and self-harm.

For our analyses, a total number of risk behaviours from 0 to 8 (BCS70) and 0 to 13 (ALSPAC) was derived for each participant. These risk behaviours are not examined individually but as a composite MRB score.

The use of the MRB measure was informed by previous research on the ALSPAC cohort that use the same configuration of health risk behaviors, which has been shown to be associated with a number of outcomes [1, 4, 5]. The individual risk behaviours were chosen based on discussions with adolescents through a young person’s research advisory group [1, 4]. The individual risk behaviours were modelled to test whether there was one behaviour that could explain the association (Supplementary File 1). We also conducted sensitivity analyses using alternative classifications of MRB, using the health risk behaviours that were individually associated with the outcomes (0–6 in BCS70, 0–7 in ALSPAC) (Supplementary File 1). These analyses, as well as previous latent class analyses using the ALSPAC cohort, did not find compelling enough evidence for an alternative classification of MRB [23].

#### Outcome: young adult SES

The outcome variable was young adult SES, which was measured by self-reported university degree attainment. Education has been found to be the most important and stable discriminator among young adults [24]. Thus, while other measures may tell us different elements of a young person’s SES, education remains a strong indicator of SES and was the primary outcome in this study.

A single question asked for highest educational attainment was asked of BCS70 participants at age 26. A series of questions were asked of ALSPAC participants between age 21 and age 24. The variable created from responses to these was binary with ‘university degree attainment’ denoting ‘high SES’.

Occupational status at age 34 was examined as a secondary outcome in the BCS70, however, these data were unavailable for ALSPAC. Occupational status was measured using the Registrar General’s Social Class classification and dichotomised, taking the lower tiers, capturing mostly manual occupations, as the ‘low SES’ reference category.

#### Early life SES variables

Parental SES was analysed using three distinct variables: maternal education, parent occupational status and household equalised income. Each variable was dichotomised into ‘high SES’ and ‘low SES’ for the purposes of comparing the association between two groups, with ‘high SES’ as the reference group as we were specifically concerned with the effect of MRB on university degree attainment for those with low SES backgrounds. Maternal education was dichotomised differently in the two birth cohorts. For BCS70, high SES denoted O-levels or higher whereas for ALSPAC, A-levels and higher were coded as high SES as it was decided this was the most relevant SES milestones within the context of the time period [25]. Few mothers in either birth cohort recorded having a degree at the time of pregnancy (2.57% (BCS70) 12.89% (ALSPAC)). A table showing the derivation of the moderator variables in both cohorts is in Supplementary File 1.

### Confounders

The analyses were adjusted for known confounders ‘sex’ and ‘conduct problems at age 10’ in both cohorts. ALSPAC had additional covariates, including ‘season of birth’ which has been shown to be an important predictor of educational attainment [4] as well as ‘IQ score at age 8 years’ and ‘key stage 2 educational attainment’ to reduce the likelihood of reverse causality between early educational performance and MRB engagement. Early life SES has been treated as a confounder in similar ALSPAC analyses, for instance between MRB and GCSE attainment [4]. However, a variable may be ‘labelled in one study as a confounder and in another study of the same outcome in the same population as a mediator or moderator, depending on which factor is the focus of each investigation’ [26]. Whereas previous studies have been concerned with adjusting for early life SES in order to remove variables that may be masking the true effect of the exposure and outcome, we are specifically interested in determining if there is a different effect between the early life SES groups.

### Statistical analysis

All analyses were done in Stata V.15. Data analysis was conducted in stages starting with descriptive statistics of the samples. Logistic regression models tested the association between adolescent MRB and young adult SES. We tested for evidence of a linear relationship between MRB and SES in young adulthood, with OR denoting reduction in odds of attaining a university degree with the increase of one health risk behaviour. Non-linearity was explored by adding quadratic terms in the MRB variable. There was no evidence that this had greater predictive power than a linear effect of the exposure on young adult SES (likelihood ratio test yielded *p* = 0.44 in BCS70 and *p* = 0.29 in ALSPAC) so the linear assumption was upheld and the imputation models developed on this basis.

Separate interactions were fitted to assess whether early life SES moderated the association between MRB (continuous) and SES in young adulthood (binary), therefore allowing the linear relationship between MRB and the log-odds for young adult SES to differ by early life SES. We hypothesised a negative effect of MRB on young adult SES. A *p*-value from the likelihood ratio test of ≤0.05 was taken as evidence of a moderation effect. This analysis was undertaken for each hypothesised moderator variable (household income, parent occupational status, maternal education) in both cohorts. We hypothesised a negative moderation effect and therefore expected an interaction term < 1. The findings from the moderation analyses are presented in tables and ‘simple slopes’ interaction plots to display the nature of the relationship interaction plot, using the *margins* and *marginsplot* commands in Stata. Moderation models are best understood using graphs or plots so that the nature of the relationship can be visualised [27]. These graphs plot the predicted values of the outcome (young adult university degree) at different levels of the exposure (adolescent MRB) using two separate lines for levels of the hypothesised moderator (early life SES). The solid line represents the predicted values of the outcome with the shaded area denoting the 95% confidence intervals around these predicted values. If the lines on the plot were parallel, this indicated there was no evidence of a moderation effect and the association between adolescent MRB and young adult degree attainment was the same for both levels of early life SES (high/low).

### Missing data

The analysis variables were presumed missing at random (MAR) as systematic differences between the observed and missing data could be explained by associations with the observed data [28]. Therefore, multiple imputation was adopted to reduce bias even though the proportion of missing data was large [29] as the MAR assumption is not determined by the amount of missingness, but rather the nature of the missingness [30]. Both birth cohorts benefited from a rich variety of auxiliary variables that were associated with missingness or the incomplete variables (BCS70: *n* = 41 ALAPC: *n* = 52). These variables were used to reduce bias, improve the precision of the imputation model and improve efficiency [4, 28]. Please see Supplementary File 1 for information on the proportion of missingness for each variable that was imputed to create our analysis sample (Supplementary Figures 1 and 2). Multiple imputation by chained equations was implemented using the *ice* command in Stata.

As the hypothesised moderator variables were categorical, we opted to stratify the dataset into high SES/low SES strata for each parental SES variable and impute separately in each stratum. This approach was motivated by the derivation of the MRB exposure variable. In order to confidently impute the exposure variable, each individual risk behaviour needed to be imputed separately and then combined (not only imputing the derived MRB score). The individual risk behaviours have been imputed separately in previous work in ALSPAC [1, 4]. Therefore, including the interaction term in the imputation model was computationally challenging with the potential for error. Undertaking the multiple imputation models in each separate stratum provided an opportunity to preserve any potential moderation effect between early life SES and the association and impute the MRB variable at the level of individual risk behaviours.

The imputation sample was those who had complete early life SES data, not the original cohorts, in order to conduct the stratified imputation approach described. For BCS70 this was *n* = 9691 (56.4%) and for ALSPAC 9001 (64.5%). Incomplete MRB (exposure), young adult SES (outcome) and incomplete confounder data were imputed up to the imputation samples.

Monte Carlo errors were used to compare the results when imputing 25, 100, 250 and 500 data sets [4] using the *mcerror* command in Stata. When calculated for the models for each of the three datasets separated by early life SES, based on 100 datasets for BSC70 and 500 datasets for ALSPAC, Monte Carlo errors were less than 10% of its standard error, with small *p* values (less than 0.01) and *t* statistic Monte Carlo errors were less than or equal to 0.1. As these three rules of thumb were upheld, reproducibility of the imputed data using 100 and 500 datasets was considered satisfactory [31]. Parameters were estimated using logistic regression in each imputed dataset. These estimates were combined by averaging across the multiple imputed datasets using Rubin’s combination rules [32]. Standard errors were calculated to show the uncertainty of the missing values, through accounting for ‘between- and within-imputation’ variation in the parameter estimates [33]. A large number of imputations was expected, given the large proportion of missing data and that a previous study on MRB in ALSPAC also required 500 imputations [4]. Complete case and imputed data were compared to assess to what extent missing data were impacting on the results. Unpaired t-tests were undertaken to compare those in the complete case samples to those with incomplete analysis variables (Supplementary File 1).

## Results

### Descriptive statistics

Of the 17,196 subjects enrolled in the birth cohort, 9691 had complete data for all three early life SES (maternal education, household income and parental occupational class) variables and therefore became the imputation sample, with all other analysis variables imputed up to this number. Of the 17,196 individuals, 8134 had all MRB (exposure) data available and 8926 had the outcome variable ‘young adult degree attainment.’ The complete case sample for the BCS70 cohort was 1358 (14.01% of the 9691 in the imputation sample and 7.90% of the birth cohort sample).

Of the 13,952 subjects included in the birth cohort (enrolled cohort, singletons and twins alive at 1 year), 9001 had all three early life SES variables (maternal education, household income and parental occupational class) recorded and this made up our imputation sample. Of the 13,952 individuals, 2656 had all MRB (exposure) data available and 5276 had the outcome variable ‘young adult degree attainment’. The complete case sample was 1360 participants (15.11% of the 9001 in the imputation sample and 9.75% of the birth cohort sample). Supplementary File 1 contains the flow diagrams describing the derivation of the imputation sample and the complete case sample for both cohorts (Supplementary Figures 3 and 4).

Table 2 contains the descriptive statistics of both samples. The descriptive statistics for the enrolled cohort samples can be found in Supplementary File 1. The median and mean number of health risk behaviours engaged in during adolescence by the complete case sample is approximately two behaviours in the BCS70 cohort (mean:1.74 (SD 1.35), median: 2 (IQR 1–3)) and three behaviours (mean: 2.91 (SD 1.89), median: 3 (IQR 1–4)) in the ALSPAC cohort. The sensitivity analysis that restricted the MRB variable to eight risk behaviours in ALSPAC revealed a lower average of behaviours comparable to the BCS70 data (mean 2.00 (SD 1.41), median: 2 (IQR 1–3).

**Table 2:** Descriptive statistics for imputed and complete case samples

	BCS70			ALSPAC		
	n	Complete case (*n* = 1358) (%)	Imputed sample (n = 9691) (SE)	n	Complete case (*n* = 1360) n (%)	Imputed sample (*n* = 9001) % (SE)
**Young Adult SES Degree attainment (outcome)**	6193			4425		
High SES		499 (36.8%)	25.5% (0.6%)		871 (64.0%)	51.8% (0.8%)
Low SES		859 (63.3%)	74.8% (0.6%)		489 (36.0%)	48.2% (0.8%)
**Young Adult SES occupation status (outcome)**	5158					
High SES		742 (54.6%)	60.5% (0.5%)		–	3.20 (0.03)
Low SES		616 (45.4%)	39.5% (0.5%)		–	–
**MRB total (exposure)**	5805			2345		
Mean (SD)		1.74 (1.4)	1.88 (0.02)		2.91 (1.9)	3.20 (0.03)
Median (IQR)		2 (1–3)	–		3 (1–4)	–
**Maternal Education SES (moderator)**	9691			9001		
High SES		567 (41.8%)	29.3% (0.5%)		697 (51.3%)	39.8% (0.5%)
Low SES		791 (58.3%)	70.7% (0.5%)		663 (48.8%)	60.2% (0.5%)
**Household Equivalised Income (moderator)**	9691			9001		
High SES (higher income brackets)		209 (15.4%)	22.5% (0.4%)		694 (51.0%)	42.1% (0.5%)
Low SES (lower income brackets)		1149 (84.6%)	77.5% (0.4%)		666 (49.0%)	57.9% (0.5%)
**Parental social class (moderator)**	9691			9001		
High SES		338 (24.9%)	25.3% (0.4%)		908 (66.8%)	57.4% (0.5%)
Low SES		1020 (75.1%)	74.7% (0.4%)		452 (33.2%)	42.6% (0.5%)
**Gender**	9691			9001		
Female		779 (57.4%)	51.7% (0.5%)		840 (61.8%)	48.8% (0.5%)
Male		579 (42.6%)	48.3% (0.5%)		520 (38.2%)	51.2% (0.5%)
**Season of birth**				9001		
Autumn	–	–	–		456 (33.5%)	33.0% (0.5%)
Winter	–	–	–		186 (13.7%)	14.1% (0.5%)
Spring	–	–	–		329 (24.2%)	23.2% (0.5%)
Summer	–	–	–		389 (28.6%)	29.7% (0.5%)
**Previous educational attainment/ability**						
IQ at age 8 Mean (SD)	–	–	–	5841	109.69 (15.0)	103.5 (0.2)
KS2 educational attainment Mean (SD)	–	–	–	6533	913.84 (154.6)	800.6 (2.6)
**Conduct problems score (age 10 years)**	9083			6007		
0		1144 (84.2%)	80.0% (0.4%)		983 (72.3%)	67.4 (0.6%)
1		178 (13.1%)	15.2% (0.4%)		316 (23.2%)	24.9 (0.6%)
2		36 (2.7%)	4.8% (0.2%)		61 (4.5%)	7.7% (0.5%)

### Associations between young adult SES and adolescent multiple risk behaviour and parental SES

#### BCS70

There was evidence of a negative association between MRB and university degree by mid-twenties (Table 3). In the BCS70, an odds ratio of 0.81 (0.76, 0.86) *p* < 0.001 shows that with each individual risk behaviour, young people have 19% reduction in odds of attaining a university degree. There was also evidence of a negative association between adolescent MRB and occupational status at age 34 (Adjusted OR 0.86 95% CI: 0.82, 0.90). There was little change in the association between adolescent MRB and young adult SES when including early life SES variables as confounders (Table 3).

**Table 3:** Associations between adolescent unit increase in MRB score and SES variables in young adulthood

	BCS70 (*N* = 9691)		ALSPAC (*N* = 9001)	
	Unadjusted OR (95% CI) *p*-value	Adjusted ^a^ OR (95% CI) *p*-value	Unadjusted OR (95% CI) *p*-value	Adjusted OR ^a^ (95% CI) *p*-value
**Outcome variables**^**b**^				
Young adult degree attainment in mid-twenties	0.80 (0.75, 0.84) *p* < 0.001	0.81 (0.76, 0.86) *p* < 0.001	0.83 (0.81, 0.86) *p* < 0.001	0.85 (0.82, 0.88) *p* < 0.001
Occupational status at age 34 years	0.84 (0.81, 0.88) *p* < 0.001	0.86 (0.82, 0.90) *p* < 0.001	–	–

^a^ Models in BCS70 were adjusted for sex and conduct score at age 10 and models in ALSPAC were adjusted for sex, IQ score age 8, conduct score at age 10, Key Stage 2 score and season born

^b^ OR are presented indicating the odds of the outcome for each incremental single behaviour out of a possible eight behaviours for BCS70 and thirteen behaviours for ALSPAC

All three parental SES variables were negatively associated with both young adult SES variables. Parents having low SES was associated with offspring not attaining a university degree. For instance, low maternal education reduced the odds of young people attaining a university degree by 62% (OR 0.38 95% CI: 0.34, 0.43). All associations in the complete case sample were comparable to those in the imputed data (Supplementary File 1).

#### ALSPAC

The direction of the association was the same in the ALSPAC cohort (Table 3), with similar odds ratios to BCS70 (Unadjusted OR: 0.83, 95% CI: 0.81, 0.86, Adjusted OR: 0.85 95% CI: 0.82, 0.88). In ALSPAC, MRB was made up of more risk behaviours (13 behaviours), however, the sensitivity analyses which modelled the association with only eight behaviours yielded similar results (Adjusted OR: 0.84, 95% CI: 0.80, 0.89).

In the ALSPAC cohort, all three early life SES variables were associated with young adult SES (university degree attainment). Parents having low SES was associated with offspring not attaining a university degree. Low maternal education reduced the odds of attaining a university degree by 51% (Adjusted OR 0.49, 95% CI: 0.42, 0.56). There was little change in the association between adolescent MRB and young adult SES when including early life SES variables as confounders (Table 3). All associations in the complete case were comparable to those in the imputed data (Supplementary File 1).

### Moderation analysis

#### BCS70

**Fig. 1:**
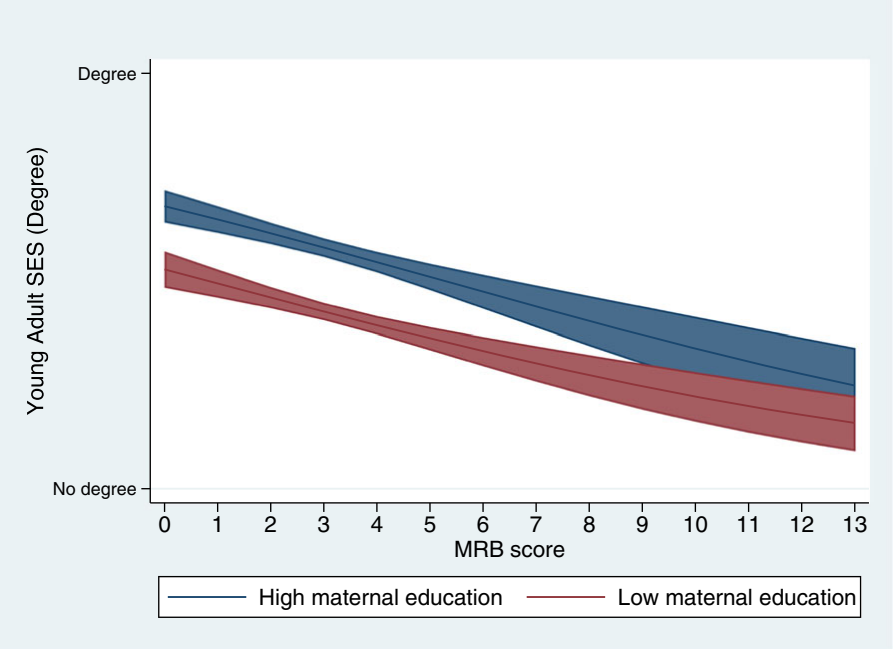
Predicted values of young adult education at each level of MRB, stratified by maternal education (*n*=9,691). Legend: Each line represents the association predicted values of the outcome (young adult degree attainment) at each level of MRB, with blue denoting the high maternal education group and red the low maternal education group. The shaded area around the lines represents the 95% confidence intervals around the predicted values

Figure 1 and Table 4 presents estimated associations between adult SES and adolescent MRB by maternal education. With increasing MRB scores, both early life SES groups had decreasing predictions of university degree attainment, however, for the low SES group the impact was greater as illustrated by the steeper gradient. Table 4 confirms that in the BCS70 sample there was evidence that maternal education (*p* = 0.03), parental occupational status (*p* = 0.009) and household income (*p* = 0.03) moderated the linear association between adolescent MRB and young adult SES (degree attainment at age 26). This means the negative effect of MRB on young adult degree attainment is stronger for those with low socioeconomic backgrounds. For the complete case sample there was evidence of a moderation effect for parental occupation only (Supplementary File 1). For the secondary outcome, there was evidence that parental occupational status moderated the relationship between adolescent MRB and occupational status at age 34 (*p* = 0.01) (Supplementary File 1).

**Table 4:** Logistic regression of young adult SES on MRB score, stratified by early life SES variables

	BCS (***n***=9691) ^**a**^				ALSPAC (***n***=9001) ^**a**^			
	All participants	High origin SES	Low origin SES	*P* value for moderation ^c^	All participants	High origin SES	Low origin SES	*P* value for moderation ^c^
**Maternal education SES**^**b**^								
MRB unit increase (0-8) and (0-13)	1 (REF)	1 (REF)	1 (REF)		1 (REF)	1 (REF)	1 (REF)	
	0.81 (0.77, 0.86) *p*<0.001	0.87 (0.80, 0.94) *p*=0.001	0.77 (0.71, 0.84) *p*<0.001	0.03	0.85 (0.82, 0.89) *p*<0.001	0.85 (0.80, 0.90) *p*<0.001	0.84 (0.81, 0.89) *p*<0.001	0.83
**Parent occupation SES**^**b**^								
MRB unit increase (0-8) and (0-13)	1 (REF)	1 (REF)	1 (REF)		1 (REF)	1 (REF)	1 (REF)	
	0.81 (0.77, 0.86) *p*<0.001	0.91 (0.83, 1.01) *p*=0.06	0.78 (0.73, 0.83) *p*<0.001	0.009	0.85 (0.82, 0.89) *p*<0.001	0.85 (0.82, 0.90) *p*<0.001	0.84 (0.79, 0.90) *p*<0.001	0.72
**Household income SES**^**b**^								
MRB unit increase (0-8) and (0-13)	1 (REF)	1 (REF)	1 (REF)		1 (REF)	1 (REF)	1 (REF)	
	0.81 (0.77, 0.86) *p*<0.001	0.87 (0.81, 0.94) *p*=0.001	0.77 (0.72, 0.84) *p*<0.001	0.03	0.85 (0.82, 0.22) *p*<0.001	0.87 (0.82, 0.92) *p*<0.001	0.85 (0.80, 0.89) *p*<0.001	0.31

^a^Models in BCS70 were adjusted for sex and conduct score at age 10 and sex, IQ score age 8, conduct score at age 10, Key Stage 2 score and season born for ALSPAC

^b^Early life SES variables are binary variables, with high SES as the reference category

^c^Likelihood ratio test *p*-values are presented, with *p* ≤0.05 taken as evidence of difference between the groups and thus a moderation effect. OR are presented indicating the odds of the outcome for each incremental single behaviour out of a possible eight behaviours for BCS70 and thirteen behaviours for ALSPAC, stratified by origin SES

#### ALSPAC

**Fig. 2:**
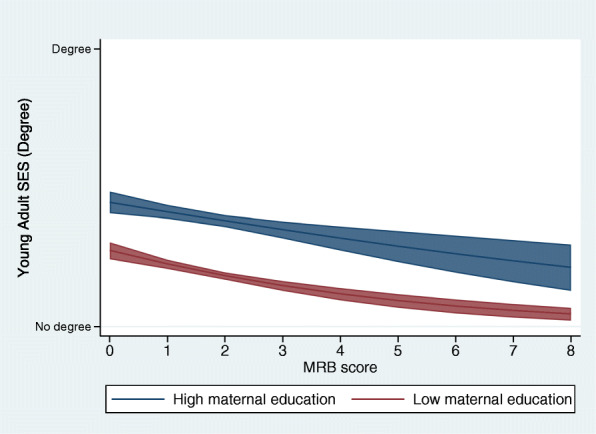
Predicted values of young adult education at each level of MRB, stratified by maternal education (*n*=9,001). Legend: Each line represents the association predicted values of the outcome (young adult degree attainment) at each level of MRB, with blue denoting the high maternal education group and red the low maternal education group. The shaded area around the lines represents the 95% confidence intervals around the predicted values

These same models applied to the imputed data in the ALSPAC cohort revealed no evidence of a moderation effect for maternal education (*p* = 0.83), parental occupation (*p* = 0.72) or household income (*p* = 0.31) (Fig. 2 and Table 4). There was no evidence of a moderation effect in the complete case (Supplementary File 1).

## Discussion

### Main findings

Adolescent MRB was negatively associated with young adult degree attainment in mid-twenties. The association was present in both cohorts. With an incremental increase of each additional risk behaviour, young people had reduced odds of attaining a degree in young adulthood. MRB was also strongly associated with occupational status at age 34 in the BCS70 cohort. There was evidence of a moderation effect in all three models for the primary outcome in the BCS70 cohort but no evidence of a moderation effect in the ALSPAC cohort. Therefore, for BCS70 participants the negative effect of adolescent MRB upon university degree attainment was stronger for young people from low socioeconomic backgrounds. For ALSPAC participants, however, the association between MRB and university degree attainment was the same regardless of early life SES.

### Strengths and limitations

This is the first longitudinal study to examine adolescent MRB and young adult SES in a UK. We also compared two birth cohorts 20 years apart and were able to derive a comparable adolescent MRB variable containing a wide range of health risk behaviours. There are several limitations. First, both cohort studies suffer from high levels of attrition. Further, non-participation and loss to follow-up is usually more pronounced among less advantaged and less healthy groups, potentially leading to bias and underestimation of inequalities [34]. To account for socially patterned attrition, we used imputed datasets derived from data collected since recruitment to maximise the chances that the assumption that observations are missing at random is satisfied. However, the method for multiple imputation may have impacted on the results. Imputing the data by early life SES stratum was necessary in order to impute each individual risk behaviour that made up the MRB variable while also preserving a potential moderation effect of early life SES on the association between MRB and young adult SES. As the early life SES variables by which the data were split were also strong predictors of the outcome variable, which had a large proportion missing, the binary outcome variable (young adult university degree) was imputed using other auxiliary variables that may not have been as informative.

A further limitation is the risk behaviours that made up the MRB variables were self-reported and reduced to binary variables, with engagement was based on cut-offs informed by the literature, which may have influenced whether an association was found [34]. The derivation of the MRB variable was based on literature and advice from young people’s groups, informing a body of MRB research in ALSPAC. There might also be differences in how the variables were reported between each cohort due to changes in data collection methods, language in questions and socially acceptable responses [16]. MRB treats all risk behaviours as equally weighted, yet outcomes may differ across the behaviours and this might be historically dependent. Reducing the number of risk behaviours in ALSPAC to the same eight behaviours in BCS70 had little effect on the regression results but did result in a lower mean and median average of risk behaviours engaged in among the ALSPAC participants.

The young adult SES variable was also self-reported as higher education data linkage was unavailable at the time of this study. It is possible that individuals aged 21 to 24 years may not have finished their education, but most will have started it given that the vast majority of individuals applying to UK universities are under age 21 [35]. Participants that were ‘currently studying’ for an undergraduate degree or for a higher degree were included in the ‘high SES’ category, given the non-continuation rate of university students by year 1 is low at 6.3% [36] and the social patterning of university applications [37]. However, this non-continuation rate is socially patterned, with a greater proportion of young people from low income backgrounds not continuing their degree beyond the first year (8.3%) [36], therefore, including these observations should be noted as a limitation of the dataset. Further, the early life SES variables were also self-reported. We chose to examine the moderators in separate analyses, but using a composite measure of SES as the moderator may have produced different results. Future research may consider this approach.

Comparability between the cohorts more generally is also limited given that BCS70 is representative of the UK, whereas ALSPAC is confined to southwest England. As the ALSPAC participants were between 21 and 24 years-old when the SES outcome variable was measured, it is possible that these young people do not yet have a settled measure of SES.

The potential impact of confounding bias should be noted as a limitation. There are a number of covariates we could have included as confounders, such as adverse childhood experiences (ACEs). However, when our study was conceptualised, we set out to include confounders consistent with a similar study in ALSPAC using MRB and an education outcome [4]. There were also limited comparable ACEs variables between the cohorts, with ALSPAC having developed an ACEs score and BCS70 using socioeconomic variables such as income and overcrowded housing as indicators of ACEs. In future studies we will also include parental risk behaviours as potential confounders, given that recent epidemiological research in ALSPAC has included these [5]. Another limitation and one potential reason for the differential results between the cohorts is the presence of residual confounding [38], which is a limitation inherent in observational epidemiology. Even large scale studies with ‘substantial adjustment may still be affected by residual confounding’ [39]. Of particular relevance to our study, the confounding effects of unmeasured genetic factors may have impacted on the results. Future research could use Mendelian Randomisation or generate polygenic scores, which has already been done in ALSPAC to investigate mental health, individual traits and substance use [40].

### Other evidence

While it has long been understood that adolescent health risk behaviours have potentially a short and long term negative impact on health (e.g. unprotected sex putting young people at risk of sexually transmitted infections, or in the long term, physical inactivity contributing to obesity later in life), there has been less research on social indicators of adult functioning such as education and employment [3]. Our finding that adolescent MRB is associated with lower educational attainment in young adulthood builds upon some of this research that has determined adolescent MRB to have negative socioeconomic outcomes later in life [3, 5, 15]. This study also mirrors findings from ALSPAC, which show a decrease in GCSE points with each additional health risk behaviour [4]. One reason this negative association might occur could be due to engagement in risk behaviours leading to disengagement in school [3] or ‘getting side-tracked’, a phrase used by one of the ALSPAC participants in the qualitative study undertaken simultaneously to this research [41]. While some engagement in health risk behaviours during adolescence may expected or even beneficial [15], our research further highlights that adolescent MRB is a public health problem that has the potential for negative consequences for young people as they enter adulthood.

The lack of evidence for a moderation effect in ALSPAC suggests that the effect of MRB is the same for young people in the cohort regardless of their SES background. This result is, however, is challenging to the wealth of evidence that people from low SES backgrounds suffer a greater burden of poor outcomes [17], as is shown in the BCS70 cohort. Furthermore, previous research has found SES to moderate the relationship between obesity and depressive symptoms in young people [42], health cognition and health behaviour [43], as well parenting practices and adolescent drinking behaviour [44]. It is also an unexpected finding given that inequalities are thought to have increased since 1970s [17]. However, the results show that early life SES is associated with young adult degree attainment, meaning if parents have low SES their children’s odds of attaining a university degree are lower. Therefore, even though there was no evidence to support the hypothesis that MRB has a greater impact on those from low SES backgrounds, it appears that there is intergenerational transmission of socioeconomic inequalities within this cohort.

Given that more young people are now attending university, one explanation for the divergent result between the cohorts is that the outcome measure of university degree attainment is not nuanced enough to explain social inequalities in contemporary young people. Therefore, while the moderating impact is less apparent for the younger cohort, there are still socioeconomic inequalities operating in terms of attendance at higher status Russell Group universities, for instance, which in turn comes with the increased likelihood of achieving a high status occupation and high income [45]. Therefore, we would be cautious of suggesting that society is more equal today than when the BCS70 participants were young adults. Instead, we propose further research to develop a measure that captures the range and complexity of young adult SES is needed to address this issue. This young adult socioeconomic measure could be a composite incorporating different income, employment and educational markers as well as subjective social status, which has been developed in the past for adolescents [10].

We know adult SES is strongly associated with a range of morbidities and mortality [17], therefore determining the impact of adolescent MRB upon young adult SES is instructive. It provides further rationale for intervening on adolescent MRB, with the aim of being more cost-effective and efficient than single behaviour interventions. Recent work has shown the success of universal school-based interventions in addressing adolescent MRB [46], thus, there is already a suite of interventions that could improve health and social outcomes.

## Conclusion

Adolescent multiple risk behaviour was negatively associated with young adult SES, measured by university degree attainment in mid-twenties and occupational status age 34. The association was similar in two birth cohorts 20 years apart. Adolescence appears to be a critical time in the life course to address risk behaviours, due to the likelihood that behaviours established here may have effects in adulthood. Each additional health risk behaviour was associated with a reduction in odds of achieving a university degree, consistent with previous work that highlighted the importance of intervening on each and every risk behaviour [4]. Evidence for a moderation effect in the BCS70 but not ALSPAC suggests that other measures should be investigated to capture the complexity of contemporary young adult SES.

## Supplementary Information

**Additional file 1.**

## Data Availability

BCS70 Cohort data comply with ESRC data sharing policies, readers can access data via the UK Data Archive (www.data-archive.ac.uk), through a formal request. This data set is open to the public. ALSPAC Data used for this submission will be made available on request to the Executive (alspac-exec@bristol.ac.uk). The ALSPAC data management plan (available here: http://www.bristol.ac.uk/alspac/researchers/data-access/documents/alspac-data-management-plan.pdf) describes in detail the policy regarding data sharing, which is through a system of managed open access. Please note that the ALSPAC study website contains details of all the data that is available through a fully searchable data dictionary and variable search tool: http://www.bristol.ac.uk/alspac/researchers/our-data/ Study data were collected and managed using REDCap electronic data capture tools hosted at University of Bristol [47]. This dataset is closed to the public, with our permission access statement appearing in the ‘ethics approval and consent to participant’ section.

## Abbreviations

MRB: Multiple Risk Behaviour
SES: Socioeconomic Status
BCS70: British Cohort Study 1970
ALSPAC: The Avon Longitudinal Study of Parents and Children
GCSE: General Certificate of Secondary Education
N: Number
CI: Confidence Interval
OR: Odds ratio
