# Correction to: Is adolescent multiple risk behaviour associated with reduced socioeconomic status in young adulthood and do those with low socioeconomic backgrounds experience greater negative impact? Findings from two UK birth cohort studies

**DOI:** 10.1186/s12889-021-11764-y

**Published:** 2021-09-30

**Authors:** Laura Tinner, Caroline Wright, Jon Heron, Deborah Caldwell, Rona Campbell, Matthew Hickman

**Affiliations:** Population Health Sciences, Bristol Medical School, University of Bristol, BG3 Oakfield House, Bristol, BS8 2BN UK

**Correction to: BMC Public Health 21, 1614 (2021)**

**https://doi.org/10.1186/s12889-021-11638-3**

In this article [1] Figs. 1 and 2 were wrongly numbered; Fig. 1 should have been Fig. 2 and vice versa as shown below. The original article has been updated.

**Fig. 1:**
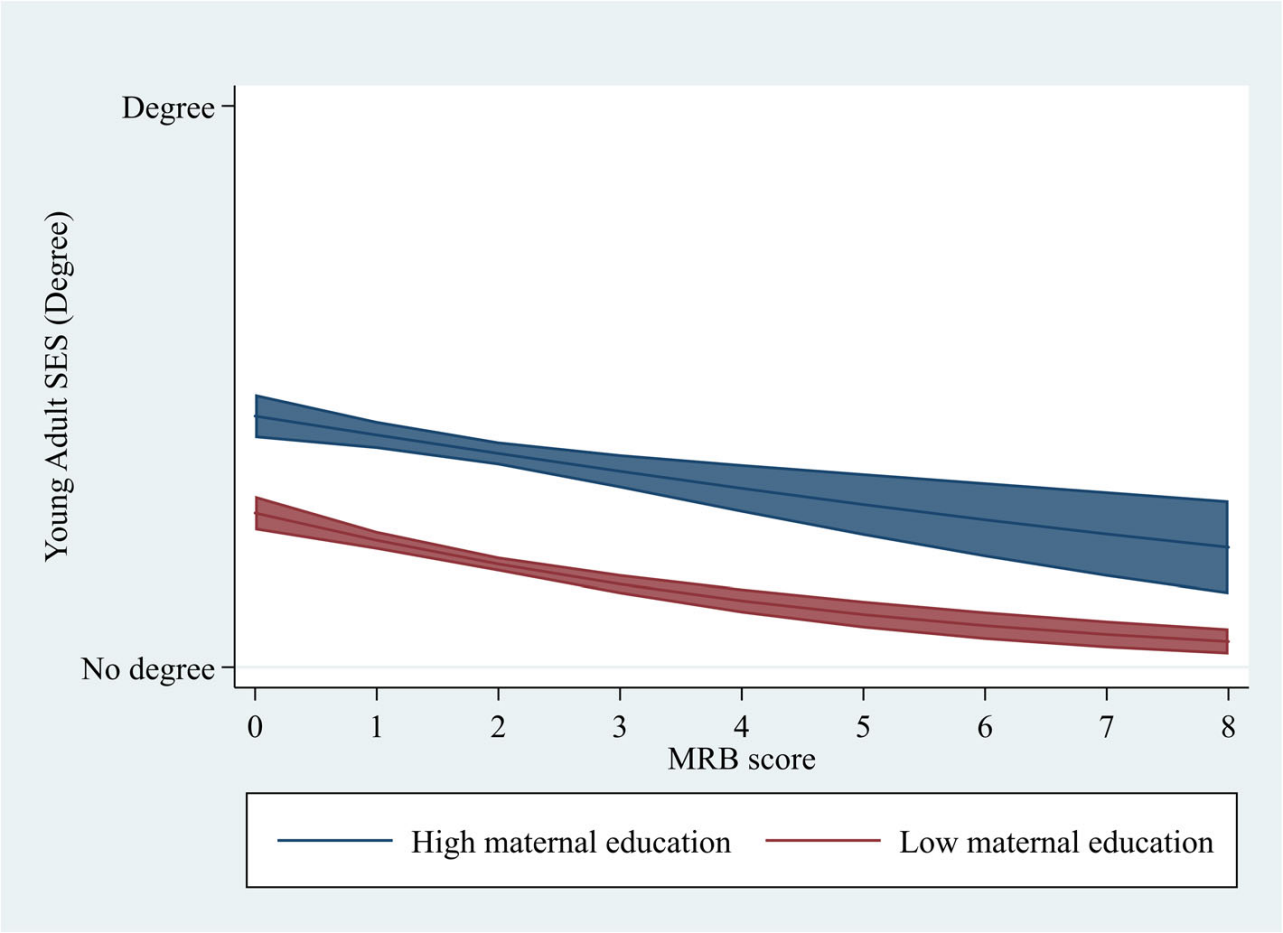
Predicted values of degree attainment at each level of MRB, stratified by maternal education (BCS70)
