# Supplementary material for: Is adolescent multiple risk behaviour associated with reduced socioeconomic status in young adulthood and do those with low socioeconomic backgrounds experience greater negative impact? Findings from two UK birth cohort studies
Source: BMC Public Health. 2021 Sep 3;21:1614. doi: 10.1186/s12889-021-11638-3 (PMC8414729; doi:10.1186/s12889-021-11638-3)
Supplement: Supplementary file 1 — Additional file 1. [file 12889_2021_11638_MOESM1_ESM.docx]

**Supplementary Material: Additional tables, figures and analyses**

**Supplementary Table 1: Derivation of the hypothesised moderator variables in both birth cohorts**

|  | **High SES** | **Low SES** |
| --- | --- | --- |
| Maternal education during pregnancy ^a^ | Attainment of O-levels or higher (BCS70)  Attainment of A-levels or higher (ALSPAC) | < O-levels (BCS70)  Attainment of O-Levels or lower (ALSPAC) |
| Household equalised income when child was age 10 years in BCS70 and age 2-4-years in ALSPAC | Top two income quintiles based on monthly income | Bottom three income quintiles based on monthly income |
| Parent occupational status during pregnancy - Registrar General’s Social Class classification ^b^ | Groups I (professional) and II (managerial/technical) | Groups III NM (skilled non-manual) and IIIM to V (skilled manual, partly skilled and unskilled) |

^a^ Maternal education is dichotomised differently in each cohort given the historical context in that in 1970 there were far fewer women who had attained O-levels or A-levels than in 1991.

^b^ For BCS70 the father’s occupation was used (or mother’s if missing) and for ALSPAC the highest occupation of either parent was used.

| **Time point** | **Variable name** | **Question** | **Response options** |
| --- | --- | --- | --- |
| Pregnancy/birth | Maternal Education | What educational qualifications do you, your partner, your mother, and your father have? Please tick all that apply. | - Degree or higher - A-levels or equivalent - O-levels or equivalent - <O-levels/no qualifications - Other |
| Pregnancy/birth | Parental Social Class | (1) Currently employed/unemployed (2) Occupation (3) Description of job (4) Self-employed/not self-employed. For BCS70 the father’s occupation was taken or mother if missing. For ALSPAC, the highest occupation of either the mother or father was taken. | Open text box or interview. ^1^ |
| Child age 10 years for BCS70. Child between 2 and 4 years for ALSPAC. | Household equivalised income^2^ | On average, about how much is the take home family income each week (include social benefits etc.)? | Quintiles of weekly family income |

**Supplementary Table 2: Questions used to derive early life SES variables in both cohorts**

^1^ The description of the job included main duties, management responsibilities, what type of company they worked for, how many employees and whether full-time/part time were coded using. Job titles and responsibilities were coded by cohort researchers using Registrar General’s Social Class classification based on occupation for the highest social class of either parent. A four-category measure was derived from the original six categories: I (professional), II (managerial/technical), IIINM (skilled non-manual) and IIIM to V (skilled manual, partly skilled and unskilled).

^2^ Income obtained was incorporated with housing and council tax benefits and then adjusted for family size and composition.

**Supplementary Table 3: Individual risk behaviour associations with the young adult degree attainment**

|  | **BCS70** | **ALSPAC** |
| --- | --- | --- |
| **Car passenger risk** | 0.75 (0.60, 0.94) p=0.01 | 0.63 (0.49, 0.80) p<0.001 |
| **Moped risk** | - | 0.56 (0.42, 0.76) p<0.001 |
| **Cycle helmet risk** | - | 0.82 (0.63, 1.05) p=0.12 |
| **Drug/solvent use** | 1.19 (.70, 2.02) p=0.52 | 0.71 (0.40, 1.28) p=0.254 |
| **Cannabis use** | 0.91 (.64, 1.29) p=0.59 | 0.63 (0.43, 0.93) p=0.02 |
| **Tobacco smoking** | 0.48 (0.40, 0.59) p<0.001 | 0.42 (0.29, 0.61) p<0.001 |
| **Hazardous alcohol use** | 0.70 (0.59, 0.84) p<0.001 | 0.80 (0.63, 1.01) p=0.06 |
| **Unprotected sex** | 0.67 (0.56, 0.82) p<0.001 | 0.25 (0.08, 0.80) p=0.02 |
| **Sex under 16 years** | - | 0.52 (0.38, 0.73) p<0.001 |
| **Self-harm** | - | 0.96 (0.73, 1.27) p=0.767 |
| **Criminal/Antisocial behaviour** | 0.68 (0.59, 0.78) p<0.001 | 0.67 (0.53, 0.84) p=0.001 |
| **Physical inactivity** | 0.84 (0.71, 0.99) p=0.04 | 0.96 (0.73, 1.24) p=0.73 |
| **TV viewing** | - | 0.80 (0.61, 1.04) p=0.12 |


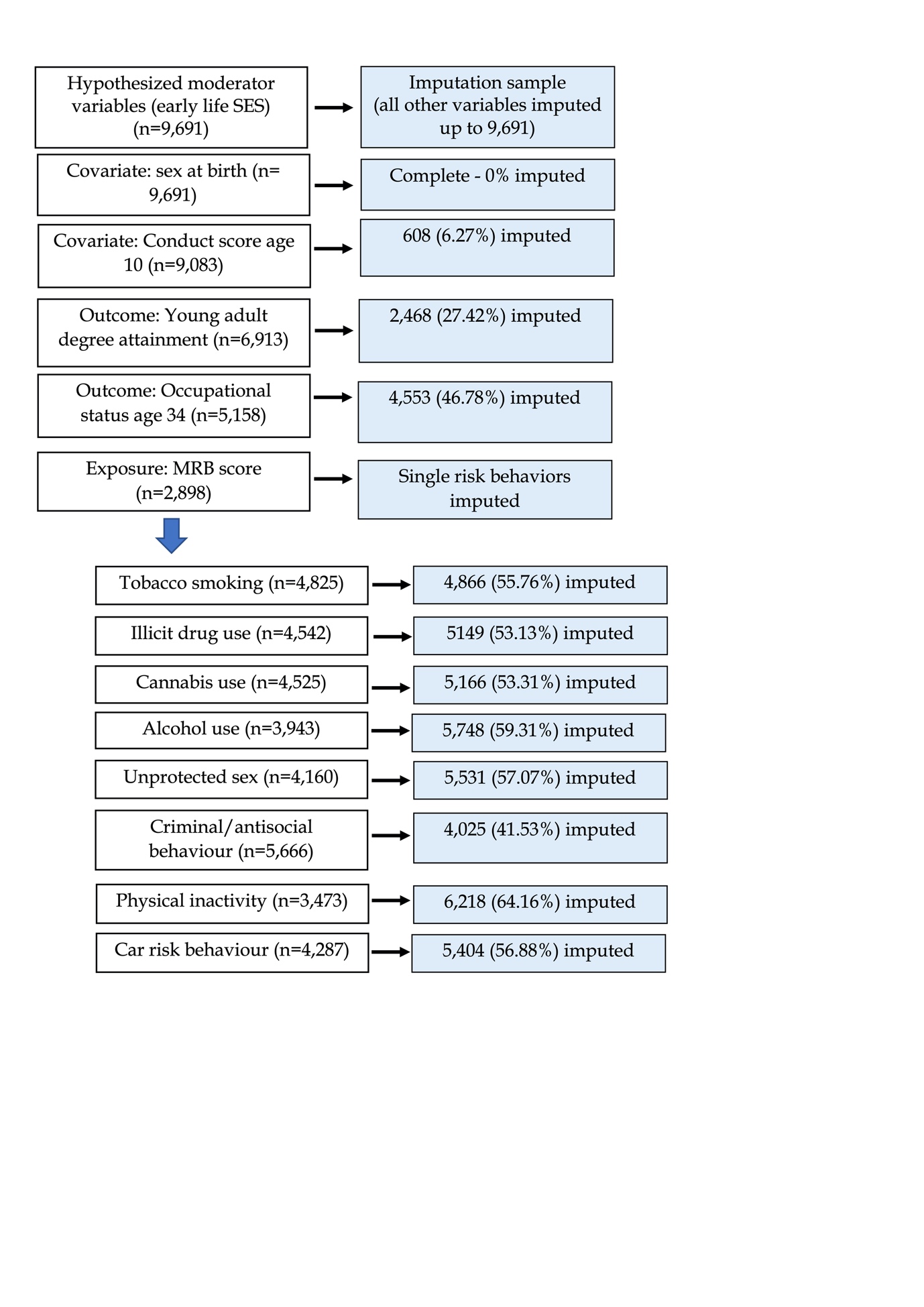


**Supplementary Figure 1: Multiple Imputation of analysis variables (BCS70)**

**Supplementary Figure 2: Multiple Imputation of analysis variables (ALSPAC)**

**Sensitivity analyses using different MRB configuration**

We conducted sensitivity analyses to assess the impact of the MRB variables as we had derived them. Firstly, we created an index of the health risk behaviours that were independently associated with the outcome. For BCS70 this was an index of 6 behaviours, with cannabis use and other drug and solvent use omitted. For ALSPAC, this index was of 7 behaviours, with TV viewing, physical inactivity, self-harm drug/solvent use and cycle helmet risk omitted.

**Supplementary Table 4: Unadjusted and adjusted associations between young adult SES (degree attainment) and MRB score (complete case and imputed sample) for seven health risk behaviours (0-7) in the ALSPAC cohort and 6 (0-6) in BCS70**

|  | **BCS70 (N=9691)** | | | **ALSPAC (N=9001)** | | |
| --- | --- | --- | --- | --- | --- | --- |
|  | Unadjusted OR (95% CI) p-value | Adjusted ^a^ OR (95% CI) p-value | Adjusted ^b^ OR (95% CI) p-value | Unadjusted OR (95% CI) p-value | Adjusted OR ^a^ (95% CI) p-value | Adjusted ^b^ OR (95% CI) p-value |
| **Outcome variables** |  |  |  |  |  |  |
| **Young adult degree attainment in mid-twenties** | 0.88 (0.84, 0.92) p<0.001 | 0.83 (0.74, 0.94) p<0.001 | 0.87 (0.80, 0.96) p<0.001 | 0.82 (0.78, 0.85) p<0.001 | 0.84 (0.80, 0.88) p<0.001 | 0.84 (0.80, 0.89)  p<0.001 |
|  |  |  |  |  |  |  |
| **Occupational status at age 34 years** | 0.92 (0.87, 0.96) p<0.001 | 0.87 (0.80, 0.95) p<0.001 | 0.88 (0.81, 0.96) p=0.005 | - | - | - |

^a^ Models in BCS70 were adjusted for sex and conduct score at age 10 and models in ALSPAC were adjusted for sex, IQ score age 8, conduct score at age 10, Key Stage 2 score and season born

^b^ Models adjusted for the confounders and also adjusted for the moderator variables maternal educational status, household equivalised income and parental occupational status.

^c^ OR are presented indicating the odds of the outcome for each incremental single behaviour out of a possible eight behaviours for BCS70 and thirteen behaviours for ALSPAC.

**Supplementary Table 5: Logistic regression of young adult SES (university degree attainment) on MRB score (0-8), stratified by early life SES variables, OR (95% CI) and interaction likelihood ratio test p-values in ALSPAC**

|  | Complete case (N=1,360) | | | | Imputed sample (N=9,001) | | | |
| --- | --- | --- | --- | --- | --- | --- | --- | --- |
|  | All participants | High origin SES | Low origin SES | P value for moderation ^c^ | All participants | High origin SES | Low origin SES | P value for moderation ^c^ |
| **Maternal education SES ^b^** |  | | | |  | | | |
| MRB (0-7) | 1 (REF) | 1 (REF) | 1 (REF) |  | 1 (REF) | 1 (REF) | 1 (REF) |  |
|  | 0.80 (0.73, 0.87) p<0.001 | 0.80 (0.71, 0.91) p=0.001 | 0.80 (0.71, 0.89) p<0.001 | 0.94 | 0.84 (0.80, 0.88) p<0.001 | 0.81 (0.75, 0.87) p<0.001 | 0.86 (0.80, 0.92) p<0.001 | 0.25 |
| **Parent occupation SES ^b^** |  | | | |  | | | |
| MRB (0-7) | 1 (REF) | 1 (REF) | 1 (REF) |  | 1 (REF) | 1 (REF) | 1 (REF) |  |
|  | 0.80 (0.74, 0.87) p<0.001 | 0.81 (0.73, 0.90) p<0.001 | 0.78 (0.68, 0.90) p=0.001 | 0.64 | 0.83 (0.78, 0.88) p<0.001 | 0.84 (0.79, 0.89) p<0.001 | 0.83 (0.76, 0.91) p<0.001 | 0.94 |
| **Household income SES ^b^** |  | | | |  | | | |
| MRB (0-7) | 1 (REF) | 1 (REF) | 1 (REF) |  | 1 (REF) | 1 (REF) | 1 (REF) |  |
|  | 0.80 (0.73, 0.87) p<0.001 | 0.77 (0.68, 0.87) p<0.001 | 0.83 (0.74, 0.93) p=0.002 | 0.38 | 0.83 (0.79, 0.88) p<0.001 | 0.84 (0.78, 0.90) p<0.001 | 0.84 (0.78, 0.89) p<0.001 | 0.97 |

^a^ Models were adjusted for IQ score age 8, conduct score at age 10, Key Stage 2 score and season born.

^b^ Early life SES variables are binary variables, with high SES as the reference category.

^c^ Likelihood ratio test p-values are presented, with p ≤0.05 taken as evidence of difference between the groups and thus a moderation effect.

OR are presented indicating the odds of the outcome for each incremental single behaviour out of a possible eight behaviours for BCS70 and thirteen behaviours for ALSPAC. Moderation of the association between adolescent MRB score and young adult SES (university degree attainment) was assessed in both cohorts for each early life SES variable, resulting in six separate models.

**Supplementary Table 6: Logistic regression of young adult SES (university degree attainment) on MRB score (0-8), stratified by early life SES variables, OR (95% CI) and interaction likelihood ratio test p-values in BCS70**

|  | Complete case (N=1,358) | | | | Imputed sample (N=9,691) | | | |
| --- | --- | --- | --- | --- | --- | --- | --- | --- |
|  | All participants | High origin SES | Low origin SES | P value for moderation ^c^ | All participants | High origin SES | Low origin SES | P value for moderation ^c^ |
| **Maternal education SES ^b^** |  | | | |  | | | |
| MRB (0-6) | 1 (REF) | 1 (REF) | 1 (REF) |  | 1 (REF) | 1 (REF) | 1 (REF) |  |
|  | 0.87 (0.80, 0.95) p<0.001 | 0.92 (0.80, 1.04) p=0.20 | 0.83 (0.75, 0.94) p=0.003 | 0.30 | 0.85 (0.78, 0.93) p<0.001 | 0.85 (0.78, 0.93) p<0.001 | 0.76 (0.69, 0.82) p<0.001 | 0.04 |
| **Parent occupation SES ^b^** |  | | | |  | | | |
| MRB (0-6) | 1 (REF) | 1 (REF) | 1 (REF) |  | 1 (REF) | 1 (REF) | 1 (REF) |  |
|  | 0.87 (0.80, 0.95) p=0.003 | 1.06 (0.90, 1.26) p=44 | 0.80 (0.72, 0.90) p<0.001 | 0.005 | 0.89 (0.79, 0.99) p=0.03 | 0.89 (0.79, 0.99) p=0.03 | 0.76 (0.71, 0.91) p<0.001 | 0.02 |
| **Household income SES ^b^** |  | | | |  | | | |
| MRB (0-6) | 1 (REF) | 1 (REF) | 1 (REF) |  | 1 (REF) | 1 (REF) | 1 (REF) |  |
|  | 0.87 (0.80, 0.96) p=0.003 | 0.87 (0.77, 0.98) p=0.02 | 0.87 (0.76, 1.00) p=0.05 | 0.99 | 0.85 (0.78, 0.92) p<0.001 | 0.85 (0.78, 0.92) p<0.001 | 0.76 (0.70, 0.82) p<0.001 | 0.06 |

^a^ Models were adjusted for IQ score age 8, conduct score at age 10, Key Stage 2 score and season born.

^b^ Early life SES variables are binary variables, with high SES as the reference category.

^c^ Likelihood ratio test p-values are presented, with p ≤0.05 taken as evidence of difference between the groups and thus a moderation effect.

OR are presented indicating the odds of the outcome for each incremental single behaviour out of a possible eight behaviours for BCS70 and thirteen behaviours for ALSPAC. Moderation of the association between adolescent MRB score and young adult SES (university degree attainment) was assessed in both cohorts for each early life SES variable, resulting in six separate models.

**Supplementary Table 7: Differences between the complete case sample and those with incomplete analysis variables**

|  | **BCS70** | | | **ALSPAC** | | |
| --- | --- | --- | --- | --- | --- | --- |
|  | Complete case sample (n= 1358) | Incomplete analysis variables (n= 8333) ^a^ | P value ^c^ | Complete case sample (n=1360) | Incomplete analysis variables (n= 7641) ^b^ | P value ^c^ |
| Male | 579 (42.64%) | 4432 (53.19%) | p<0.001 | 520 (38.24%) | 4087 (53.49%) | p<0.001 |
| Free school meal eligibility | 104 (7.66%) | 1354 (16.42%) | p<0.001 | 49 (3.60%) | 691 (9.04%) | p<0.001 |
| Achieved 5 or more GCSEs grade A*-C | - | - | - | 1169 (85.96%) | 3143 (51.33%) | p<0.001 |
| Maternal education – high SES | 567 (41.45%) | 2277 (27.33%) | p<0.001 | 697 (51.25%) | 2886 (37.77%) | p<0.001 |
| Parent occupation – high SES | 338 (24.89%) | 1355 (16.26%) | p<0.001 | 908 (66.76%) | 4259 (55.74%) | p<0.001 |
| Household income – high SES | 487 (35.86%) | 2457 (29.49%) | p<0.001 | 694 (51.03%) | 3092 (40.47%) | p<0.001 |

^a^ Participants with incomplete exposure, outcome and covariate data but complete early life socioeconomic data and sex at birth. The complete case sample (1,358) added to those with incomplete analysis variables (8,333) equates to the imputed sample (9,691).

^b^ Participants with incomplete exposure, outcome and covariate data but complete early life socioeconomic data and sex at birth. The complete case sample (1,360) added to those with incomplete analysis variables (7,641) equates to the imputed sample (9,001).

^c^ p-value from unpaired t-test assessing difference between the included and excluded groups.

**Supplementary Figure 3: Complete case sample derivation - BCS 1970**

Enrolled cohort

(n = 17,196)

Participants from Northern Ireland not followed beyond birth (n=628)

n= 9,691

n=5,154

n=1,409

Complete Case sample

(n =1,358)

Missing SES variables: maternal education, parental occupational class and household equivalised income (n=6,877)

Missing outcome variables: young adult education and occupation at age 34 (n=4,537)

Missing exposure variable: Multiple risk behaviour score (n=3,745)

n=16,568

Missing covariate data (sex and conduct score age 10) (n=51)

**Supplementary Figure 4: Complete case derivation for ALSPAC**

Enrolled cohort, singletons and twins alive at 1 year (n = 13952)

Missing SES variables: maternal education, parental occupational class and household equivalised income (n=4951)

n= 9001

n=4425

n=2055

Complete Case sample

(n = 1360)

Missing outcome variable: young adult education (n=4576)

Missing exposure variable: Multiple risk behaviour score (n= 2370)

Missing covariate data (IQ age 8, conduct score age 10, Key Stage 2 grade) (n=695)

|  | BSC70 (n=17,196) | | ALSPAC (n=13,952) | |
| --- | --- | --- | --- | --- |
|  | n^a^ | (%) | n^a^ | (%) |
| **Young Adult SES Degree attainment (outcome)** | 8,926 |  | 5,276 |  |
| High SES |  | 2,401 (26.90%) |  | 2,928 (55.50%) |
| Low SES |  | 6,525 (73.10%) |  | 2,348 (55.50%) |
| **Young Adult SES occupation status (outcome)** | 7,416 |  |  |  |
| High SES |  | 3,440 (52.61%) |  | - |
| Low SES |  | 3,976 (46.39%) |  | - |
| **MRB total (exposure)** | 8,134 |  | 2,656 |  |
| Mean (SD) |  | 1.40 (1.28) |  | 2.96 (1.98) |
| Median (IQR) |  | - |  | - |
| **Maternal Education SES (moderator)** | 12,179 |  | 12,393 |  |
| High SES |  | 8,662 (71.12%) |  | 4,383 (35.37%) |
| Low SES |  | 3,517 (28.88%) |  | 8,010 (64.63%) |
| **Household Equivalised Income (moderator)** | 11,678 |  | 9,918 |  |
| High SES (higher income brackets) |  | 8,156 (69.84%) |  | 3,989 (40.22%) |
| Low SES (lower income brackets) |  | 3,522 (30.16%) |  | 5,929 (59.78%) |
| **Parental social class (moderator)** | 17,061 |  | 11,476 |  |
| High SES |  | 13,951 (81.77%) |  | 6,318 (55.05%) |
| Low SES |  | 3,110 (18.23%) |  | 5,158 (44.95%) |
| **Gender** | 17,185 |  | 13,952 |  |
| Female |  | 8,279 (48.18%) |  | 6,747 (48.36%) |
| Male |  | 8,906 (51.82%) |  | 7,205 (51.64%) |
| **Season of birth** |  |  | 13,952 |  |
| Autumn | - | - |  | 4,683 (33.57%) |
| Winter | - | - |  | 1,955 (14.01%) |
| Spring | - | - |  | 3,157 (22.63%) |
| Summer | - | - |  | 4,157 (29.80%) |
| **Previous educational attainment/ability** |  |  |  |  |
| IQ at age 8 Mean (SD) | - | - | 7,057 | 104.14 (16.50) |
| KS2 educational attainment Mean (SD) | - | - | 9,752 | 799.82 (182.39) |
| **Conduct problems score (age 10 years)** | 11,933 |  | 7,027 |  |
| 0 |  | 9,555 (80.07%) |  | 4,770 (67.88%) |
| 1 |  | 1,795 (15.04%) |  | 1,738 (24.73%) |
| 2 |  | 583 (4.89%) |  | 519 (7.39%) |

**Supplementary Table 8: Descriptive statistics for initial enrolled cohort samples**

^a^ N refers to individuals who answered the questionnaire or clinic questions for each variable and therefore have complete data for that variable. For example, for BSC70, 8,926 participants out of the initially enrolled cohort sample (n=17,196) have university degree attainment data.

**Supplementary Table 9: Associations between adolescent unit increase in MRB score and SES variables (complete case)**

|  | | **BCS70 (N=1358)** | | **ALSPAC (N=1360)** | |
| --- | --- | --- | --- | --- | --- |
|  |  | Unadjusted OR (95% CI) p-value | Adjusted ^a^ OR (95% CI) p-value | Unadjusted OR (95% CI)  p-value | Adjusted OR ^a^ (95% CI) p-value |
| **Exposure variable** | |  |  |  |  |
| Multiple risk behaviour (0-8 and 0-10) ^b^ | | 0.87 (0.80, 0.94) p=0.001 | 0.87 (0.80, 0.95) p=0.002 | 0.84 (0.79, 0.89) p<0.001 | 0.85 (0.80, 0.90) p<0.001 |
| **Early life SES variables** ^c^ | |  |  |  |  |
| Maternal education | High SES | 1 (REF) | 1 (REF) | 1 (REF) | 1 (REF) |
|  | Low SES | 0.51 (0.41, 0.64) p=0.002 | 0.51 (0.41, 0.64) p<0.001 | 0.38 (0.35, 0.43) p<0.001 | 0.58 (0.45, 0.74) p<0.001 |
| Parent occupational status | High SES | 1 (REF) | 1 (REF) | 1 (REF) | 1 (REF) |
|  | Low SES | 0.37 (0.29, 0.48) p<0.001 | 0.38 (0.29, 0.49) p<0.001 | 0.50 (0.40, 0.64) p<0.001 | 0.63 (0.49, 0.80) p<0.001 |
| Household income | High SES | 1 (REF) | 1 (REF) | 1 (REF) | 1 (REF) |
|  | Low SES | 0.56 (0.44, 0.70) p<0.001 | 0.56 (0.45, 0.71) p<0.001 | 0.57 (0.46, 0.72) p<0.001 | 0.74 (0.58, 0.94) p=0.01 |

^a^ Models in BCS70 were adjusted for sex and conduct score at age 10 and sex, IQ score age 8, conduct score at age 10, Key Stage 2 score and season born for ALSPAC.

^b^ OR are presented indicating the odds of the outcome for each incremental single behaviour out of a possible eight behaviours for BCS70 and ten behaviours for ALSPAC as ten was the maximum number of behaviours engaged in for the complete case participants.

^c^ Early life SES variables are binary variables, with high SES as the reference category.

**Supplementary Table 10: Moderation analyses in complete case samples**

|  | **BCS70 (n=1358) ^a^** | | | | **ALSPAC (n=1360) ^a^** | | | |
| --- | --- | --- | --- | --- | --- | --- | --- | --- |
|  | All participants | High origin SES | Low origin SES | P value for moderation ^c^ | All participants | High origin SES | Low origin SES | P value for moderation ^c^ |
| **Maternal education SES ^b^** |  | | | |  | | | |
| Unit increase in MRB (0-8) (0-10) | 1 (REF) | 1 (REF) | 1 (REF) |  | 1 (REF) | 1 (REF) | 1 (REF) |  |
|  | 0.88 (0.80, 0.95) p=0.003 | 0.92 (0.81, 1.04) p=0.168 | 0.84 (0.75, 0.95) p=0.005 | 0.34 | 0.85 (0.80, 0.91) p<0.001 | 0.87 (0.79, 0.96) p=0.004 | 0.84 (0.77, 0.92) p<0.001 | 0.60 |
| **Parent occupation SES ^b^** |  | | | |  | | | |
| Unit increase in MRB (0-8) (0-10) | 1 (REF) | 1 (REF) | 1 (REF) |  | 1 (REF) | 1 (REF) | 1 (REF) |  |
|  | 0.88 (0.81, 0.96) p=0.003 | 1.05 (0.90, 1.24) p=0.49 | 0.81 (0.72, 0.90) p<0.001 | 0.006 | 0.85 (0.80, 0.91) p<0.001 | 0.85 (0.79, 0.92) p<0.001 | 0.85 (0.77, 0.94) p=0.003 | 0.98 |
| **Household income SES ^b^** |  | | | |  | | | |
| Unit increase in MRB (0-8) (0-10) | 1 (REF) | 1 (REF) | 1 (REF) |  | 1 (REF) | 1 (REF) | 1 (REF) |  |
|  | 0.88 (0.80, 0.95) p=0.002 | 0.90 (0.78, 1.03) p=0.130 | 0.86 (0.77, 0.96) p=0.007 | 0.63 | 0.85 (0.80, 0.91) p<0.001 | 0.85 (0.77, 0.93) p<0.001 | 0.86 (0.78, 0.93) p<0.001 | 0.92 |

^a^ Models in BCS70 were adjusted for sex and conduct score at age 10 and sex, IQ score age 8, conduct score at age 10, Key Stage 2 score and season born for ALSPAC.

^b^ Early life SES variables are binary variables, with high SES as the reference category.

^c^ Likelihood ratio test p-values are presented, with p ≤0.05 taken as evidence of difference between the groups and thus a moderation effect.

OR are presented indicating the odds of the outcome for each incremental single behaviour out of a possible eight behaviours for BCS70 and thirteen behaviours for ALSPAC.

Moderation of the association between adolescent MRB score and young adult SES (university degree attainment) was assessed in both cohorts for each early life SES variable, resulting in six separate models. Results are presented as odds ratios for all participants and for the two SES subgroups. The maximum MRB score in BCS70 complete case is 8 and in ALSPAC complete case is 10.

**Supplementary Table 11: Moderation analyses for occupational status age 34 outcome in BCS70**

|  | **Complete case BCS70 (n=1358) ^a^** | | | | **Imputed data BCS70 (n=9691)** | | | |
| --- | --- | --- | --- | --- | --- | --- | --- | --- |
|  | All participants | High origin SES | Low origin SES | P value for moderation | All participants | High origin SES | Low origin SES | P value for moderation |
| **Maternal education SES ^b^** |  | | | |  | | | |
| Unit increase in MRB (0-8) | 1 (REF) | 1 (REF) | 1 (REF) |  | 1 (REF) | 1 (REF) | 1 (REF) |  |
|  | 0.89 (0.82, 0.96) p=0.003 | 0.91 (0.81, 1.04) p=0.188 | 0.86 (0.78, 0.96) p=0.007 | 0.48 | 0.86 (0.82, 0.90) p<0.001 | 0.88 (0.81, 0.95) p=0.002 | 0.85 (0.80, 0.89) p<0.001 | 0.41 |
| **Parent occupation SES ^b^** |  | | | |  | | | |
| Unit increase in MRB (0-8) | 1 (REF) | 1 (REF) | 1 (REF) |  | 1 (REF) | 1 (REF) | 1 (REF) |  |
|  | 0.89 (0.82, 0.96) p=0.004 | 1.03 (0.87, 1.23) p=0.73 | 0.85 (0.77, 0.93) p<0.001 | 0.05 | 0.85 (0.81, 0.89) p<0.001 | 0.98 (0.87, 1.09) p=0.68 | 0.82 (0.77, 0.87) p<0.001 | 0.012 |
| **Household income SES ^b^** |  | | | |  | | | |
| Unit increase in MRB (0-8) | 1 (REF) | 1 (REF) | 1 (REF) |  | 1 (REF) | 1 (REF) | 1 (REF) |  |
|  | 0.88 (0.81, 0.96) p=0.003 | 0.97 (0.84, 1.11) p=0.629 | 0.84 (0.77, 0.94) p=0.001 | 0.14 | 0.85 (0.80, 0.89) p<0.001 | 0.90 (0.83, 0.97) p=0.007 | 0.82 (0.77, 0.88) p<0.001 | 0.12 |

^a^ Models in BCS70 were adjusted for sex and conduct score at age 10

^b^ Early life SES variables are binary variables with derivation shown in Table 1.

^c^ Likelihood ratio test p-values are presented, with p ≤0.05 taken as evidence of difference between the groups and thus a moderation effect.

OR are presented indicating the odds of the outcome for each incremental single behaviour out of a possible eight behaviours for BCS70 and thirteen behaviours for ALSPAC. Moderation of the association between adolescent MRB score and young adult SES (occupational status at age 34) was assessed in the BCS70 cohorts for each early life variable, resulting in three separate models. Results are presented as odds ratios for all participants and for the two SES subgroups. The exposure variable (MRB) is continuous, with odds ratios indicating the effect of one risk behaviour on the outcome (young adult SES). Likelihood ratio test p-values are presented, with p ≤0.05 taken as evidence of difference between the groups and thus a moderation effect
